# Supplementary material for: Orbital character of the spin-reorientation transition in TbMn6Sn6
Source: Nat Commun. 2023 May 9;14:2658. doi: 10.1038/s41467-023-38174-5 (PMC10169834; doi:10.1038/s41467-023-38174-5)
Supplement: Supplementary file 1 — Supplementary Information [file 41467_2023_38174_MOESM1_ESM.pdf]

Supplementary Information - Orbital character of the spin-reorientation transition in $TbMn_6Sn_6$

S. X. M. Riberolles,[1] Tyler J. Slade,[1,2] R. L. Dally,[3] P. M. Sarte,[4] Bing Li,[2] Tianxiong Han,[2] H. Lane,[5,6] C. Stock,[5] H. Bhandari,[7,8] N. J. Ghimire,[7,8] D. L. Abernathy,[9] P. C. Canfield,[1,2] J. W. Lynn,[3] B. G. Ueland,[1] and R. J. McQueeney[1,2]

[1]*Ames National Laboratory, Ames, Iowa, 50011, USA*

[2]*Department of Physics and Astronomy, Iowa State University, Ames, Iowa, 50011, USA*

[3]*NIST Center for Neutron Research, National Institute of Standards and Technology, Gaithersburg, Maryland 20899, USA*

[4]*Materials Department and California Nanosystems Institute, University of California Santa Barbara, Santa Barbara, California 93106, USA*

[5]*School of Physics and Astronomy, University of Edinburgh, Edinburgh EH9 3JZ, United Kingdom*

[6]*School of Physics, Georgia Institute of Technology, Atlanta, Georgia 30332, USA*

[7]*Department of Physics and Astronomy, George Mason University, Fairfax, VA 22030, USA*

[8]*Quantum Science and Engineering Center, George Mason University, Fairfax, VA 22030, USA*

[9]*Neutron Scattering Division, Oak Ridge National Laboratory, Oak Ridge, Tennessee 37831 USA*

I. SUPPLEMENTARY NOTE 1: DETERMINATION OF MAGNETIC HAMILTONIAN PARAMETERS

At $T = 7$ K, we fit the INS dispersion with linear spin-wave theory (LSWT) using the `SpinW` package [1]. We fit to a minimal description of the magnetic Hamiltonian with simplified single-ion anisotropy terms $\mathcal{H}_{\mathrm{Tb}} + \mathcal{H}_{\mathrm{Mn}} = D^T \sum_j (J_z)_j^2 + D^M \sum_i (s_z)_i^2$. In reference [2], the Mn-Tb coupling is described as a coupling between the Mn and Tb *spins*, as is typically expected from RKKY or superexchange type interactions. For Tb166, this model has a demonstrated deficiency in that it cannot account for the observed spin gap without introducing a large *uniaxial* Mn single-ion anisotropy, a result that is not consistent with the observation of easy-plane magnetism in YMn_6Sn_6 and $GdMn_6Sn_6$. A description where coupling occurs between the spin of Mn and the *total* angular momentum, J, of the Tb ion can equally well fit the inelastic neutron data. The fitting results to the centroid (in energy) of various peaks are shown in Fig. 3 and we obtain the parameters listed in Table 1. This fitting confirms the strong uniaxial single-ion anisotropy for Tb ($D^T < 0$) and easy-plane anisotropy for Mn ($D^M > 0$), as expected, and provides much better agreement with the spin gap and the Tb magnetic anisotropy constants reported in the literature.

Note that, in *SpinW*, it is generally not possible to map the exchange coupling parameters into an appropriate spin-only LSWT model. Only in RPA theory can exchange coupling through the spin be handled appropriately, since it can explicitly include the spin-orbit coupling. Perhaps counterintuitively, introducing coupling through J is the appropriate effective model when using LSWT to fit systems composed of mixed rare-earth and transition metal ions. An appropriate set of LWST parameters can be obtained using a model where exchange coupling occurs through J by implementing an effective exchange $\mathcal{J}'^{MT} = (g - 1)\mathcal{J}^{MT}$, where $g = 3/2$ is the Lande-g factor for Tb. Using this scaling, the resulting LSWT excitation spectra are numerically consistent with RPA analysis where coupling through the Tb spin is properly implemented. The ground state RPA calculations, `SpinW`, and `UppASD` packages reproduce the correct spin wave dispersions.

Supplementary Table 1. Linear spin-wave theory model parameters and CEF parameters (in meV) obtained from analysis of INS data in the ground state of $TbMn_6Sn_6$.

$\mathcal{J}^{MT}$	$\mathcal{J}_0^{MM}$	$\mathcal{J}_1^{MM}$	$\mathcal{J}_2^{MM}$	$\mathcal{J}_3^{MM}$	D^T	D^M	B_2^0	B_4^0
1.83 (3)	-28.8 (1)	-4.4(4)	-19.2 (2)	1.8 (2)	-1.28 (2)	0.44(6)	-0.0347	-0.00143

II. SUPPLEMENTARY NOTE 2: TB LOCAL-ION HAMILTONIAN

The local-ion Hamiltonian of the Tb ion consists of the CEF potential and the molecular field generated at the Tb site from exchange coupling to Mn,

$$\mathcal{H}_{\mathrm{Tb}} + \mathcal{H}_{MF} = B_2^0 \mathcal{O}_2^0 + B_4^0 \mathcal{O}_4^0 - 12\mathcal{J}^{MT}\langle s_z\rangle S_z. \qquad (1)$$

The $\mathcal{O}_l^0$ depend only on powers of the angular momentum projection operator J_z^l of the $J = 6$ Tb ion. Thus, the eigenstates of $\mathcal{H}_{\mathrm{Tb}}$ are also eigenstates of J_z, $|\pm m_J\rangle$. The ground state magnetic anisotropy energy (MAE) of the Tb is $E_{MAE} = K_1 \sin^2\theta + K_2 \sin^4\theta$, where $K_1 = -3B_2^0 J^{(2)} - 40B_4^0 J^{(4)}$ and $K_2 = 35B_4^0 J^{(4)}$ and $J^{(2)} = 33$ and $J^{(4)} = 742.5$ for the $J = 6$ Tb ion. The SR-transition is enabled by a large and negative K_2, which lowers the in-plane MAE.

To determine experimental values of B_2^0 and B_4^0, we first note that the single-ion anisotropy parameter obtained from INS data is related to the first-order magnetic anisotropy energy parameter $K_1 = -D^T J^2 = 46$ meV. The second-order parameter $K_2 = 35B_4^0 J^{(4)}$ may be estimated by assuming that the SR-transition occurs when $K_1(T) + K_2(T) + 6s^2 D^M \approx 0$ at $T_{SR} \approx 310$ K. $K_n(T)$ are obtained from the thermal averages of the Stevens operators according to $K_1(T) = -3B_2^0\langle\mathcal{O}_2^0\rangle/2 - 5B_4^0\langle\mathcal{O}_4^0\rangle$ and $K_2(T) = 35B_4^0\langle\mathcal{O}_4^0\rangle/8$, as shown in Fig. 4. Using this approach, we obtain $K_2 = -38.4$ meV, $B_2^0 = -0.035$, and $B_4^0 = -0.00143$ meV [as indicated by the orange arrow in Fig. 4(a) of the main text]. These CEF parameters compare reasonably well with DFT [3] and magnetization data [4, 5].

At low temperatures, the molecular field $12\mathcal{J}^{MT}\langle s_z\rangle$ is parallel to the c-axis (proportional to J_z) and therefore diagonal in the $|J, m_J\rangle$ basis. It will cause a Zeeman splitting of the Tb $|\pm m_J\rangle$ CEF states, but not mix them. In the SR-phase, the molecular field is in the xy-plane and will mix the J_z states, leading to a change in the level spectra and matrix elements.

III. SUPPLEMENTARY NOTE 3: FREE-ENERGY CALCULATIONS OF THE SR-TRANSITION

We calculate the free energy of the magnetic Hamiltonian within the mean field approximation,

$$\begin{aligned}\mathcal{F} = &- k_B T \ln(Z_{\rm Tb}) - 6 k_B T \ln(Z_{\rm Mn}) \\ &- 12\mathcal{J}^{MT}\langle \mathbf{s}\rangle \cdot \langle \mathbf{S}\rangle - 3\sum_i \gamma_i \mathcal{J}_i^{MM}\langle \mathbf{s}_i\rangle \cdot \langle \mathbf{s}\rangle.\end{aligned} \tag{2}$$

Here, $Z_{\rm Tb}$ and $Z_{\rm Mn}$ are the partition functions of Tb and Mn ions in the crystal-field plus molecular-field local Hamiltonian, as in Eqn. S1. The last two terms are the average exchange energy of the system and γ_i is the number of neighbors of a given exchange path.

We use the Heisenberg parameters above and constrain the Tb and Mn sublattice spins to lie in a vertical plane with θ_T and θ_M representing the angle of the moments from the c-axis. In the uniaxial ferrimagnetic phase, $\theta_T = 180^{\rm o}$ and $\theta_M = 0$ while the easy-plane state corresponds to $\theta_T = \theta_M = 90^{\rm o}$. In both cases the sublattices remain ferrimagnetic with $\langle \mathbf{S}\rangle = -\langle \mathbf{s}\rangle$ and the thermally-averaged spins are determined self-consistently. Figure 5 (a) shows that the uniaxial ferrimagnet is the equilibrium state at low temperatures and there is a transition to the easy-plane ferrimagnet above $T_{SR} \approx 310$ K, in correspondence with similar calculations of the MAE shown in Fig. 4. Fig. 5(b) shows the energy landscape as a function of the Mn angle for several temperatures. The free energy shows first-order character whereby local minima are maintained at $\theta_M = 0$ and $90^{\rm o}$ with a discontinuous change in the global minimum at T_{SR}.

[1] Toth, S. & Lake B. Linear spin wave theory for single-Q incommensurate magnetic structures. *J. Phys.: Condens. Matter* **27**, 166002 (2015).

[2] Riberolles, S. X. M. et al. Low-Temperature Competing Magnetic Energy Scales in the Topological Ferrimagnet $TbMn_6Sn_6$. *Phys. Rev. X* **12**, 021043 (2022).

[3] Lee, Y. et al. Interplay between magnetism and band topology in Kagome magnets RMn_6Sn_6. Preprint at http://arXiv.org/abs/2201.11265 (2022).

[4] Guo, G.-H. & Zhang, H.-B. The spin reorientation transition and first-order magnetization process of $TbMn_6Sn_6$ compound. *J. Alloys Compd.* **448**, 17-20 (2008).

[5] Zajkov, N. K., Mushnikov, N. V., Bartashevich, M. I. & Goto, T. Magnetization processes in the $TbMn_6Sn_6$ compound. *J. Alloys Compd.* **309**, 26-30 (2000).

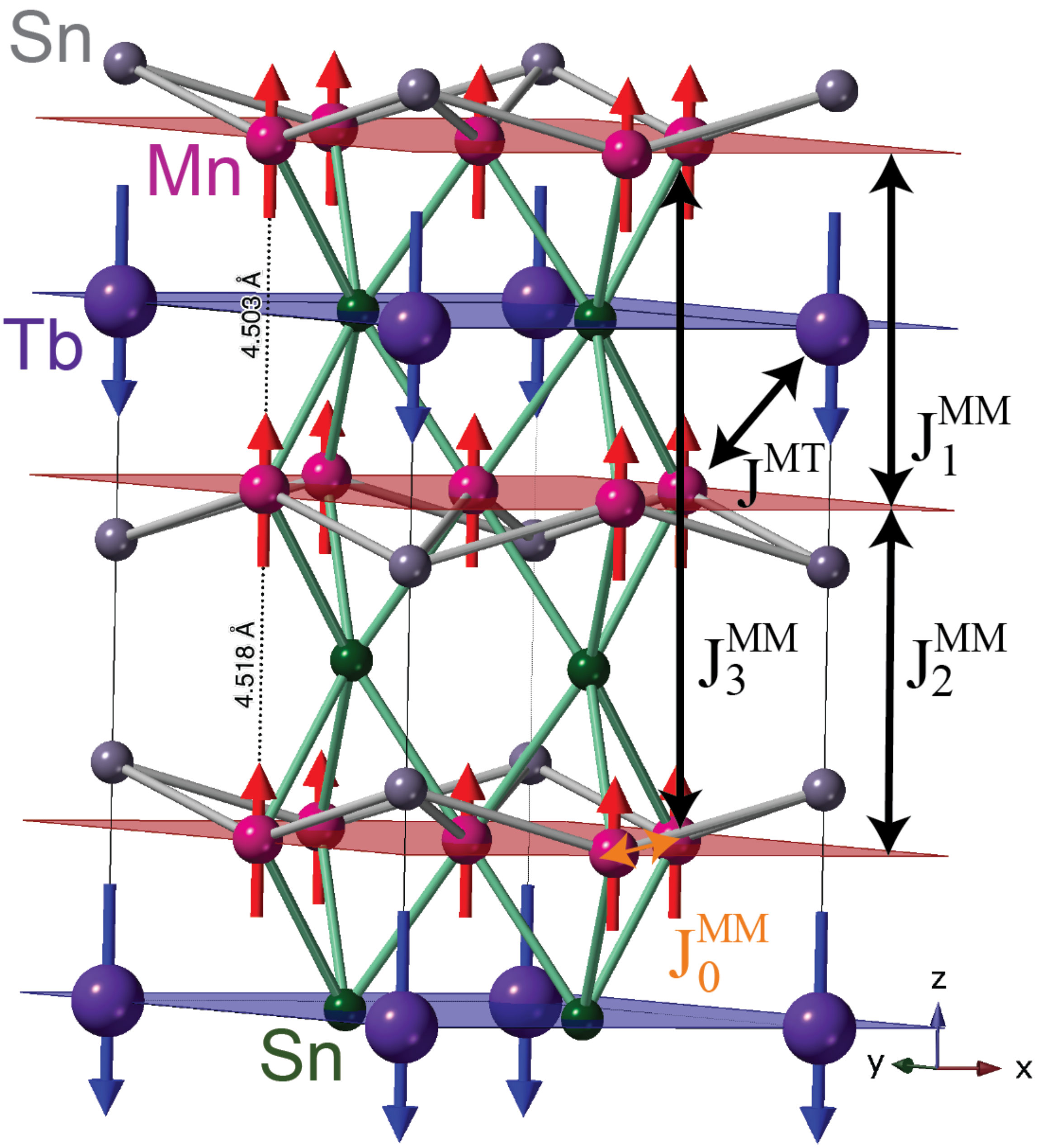

Supplementary Figure 1. **Crystal and magnetic structure of $TbMn_6Sn_6$.** The low-temperature uniaxial ferrimagnetic structure of $TbMn_6Sn_6$ with magnetic moments indicated by arrows. Black two-headed arrows label the key magnetic interactions along the c-axis. The in-plane Mn-Mn interaction (orange arrow) is strong and ferromagnetic. The in-plane Tb-Tb exchange is small and has been neglected.

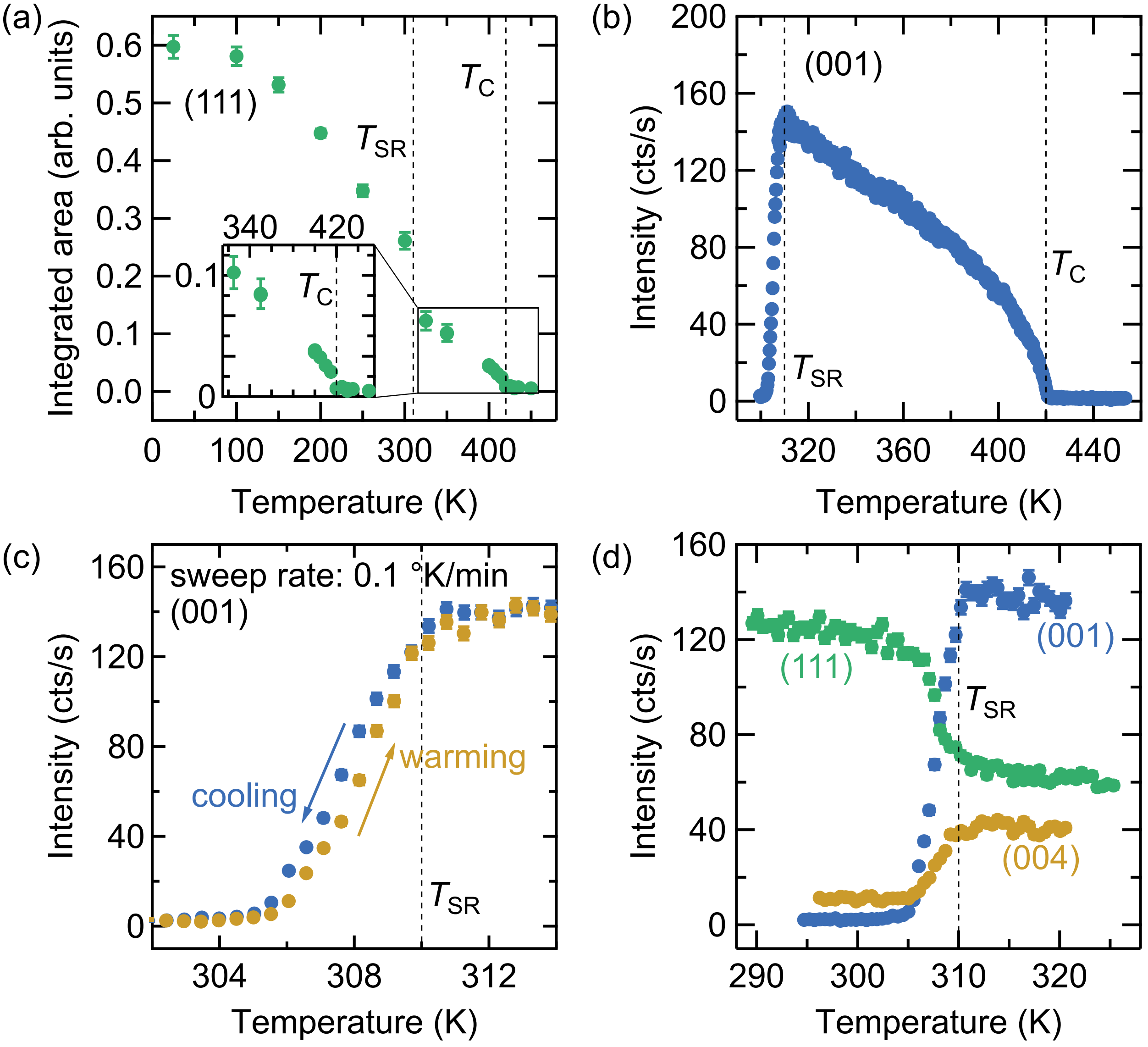

Supplementary Figure 2. **Neutron diffraction from $TbMn_6Sn_6$ measured on BT-7.** Temperature dependence of the (1,1,1) Bragg peak integrated intensity (a) over a large temperature range and (inset) zoomed in to the ordering transition at T_C. Data were taken by rotating the sample about the (1,1,1) Bragg peak reflection. (b) Temperature evolution of the (0,0,1) Bragg peak intensity showing T_C and T_{SR} and taken while cooling. (c) Cooling and warming data of the (0,0,1) Bragg peak intensity taken with a slow temperature sweep magnitude of 0.1 K/min. (d) Temperature evolution of the (0,0,1), (0,0,4), and (1,1,1) Bragg peak intensities near T_{SR} taken while cooling. Error bars indicate plus and minus one standard deviation.

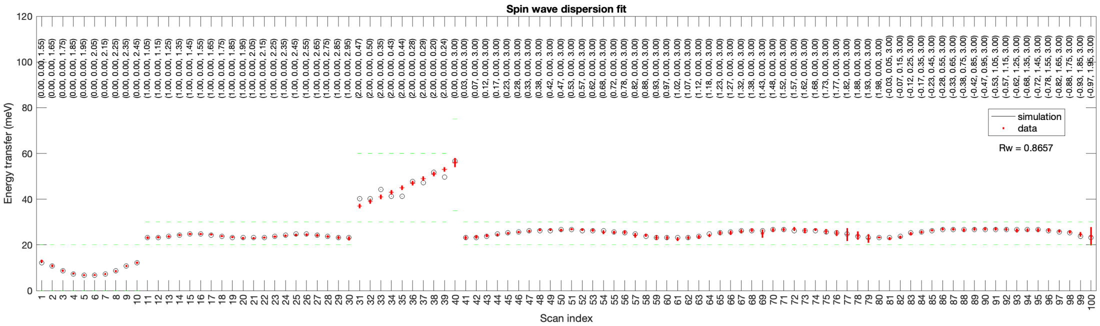

Supplementary Figure 3. **Fits to the spin low-energy spin wave dispersion of $TbMn_6Sn_6$ using linear spin wave theory**. The energy of different spin waves labeled by the **q**-vector were determined from gaussian fits to constant-**q** energy cuts (red symbols). Error bars are one standard deviation, as determined from least-squares fit to gaussian lineshapes. The open symbols are the energy values determined by a best fit to the Heisenberg hamiltonian as described in the main text.

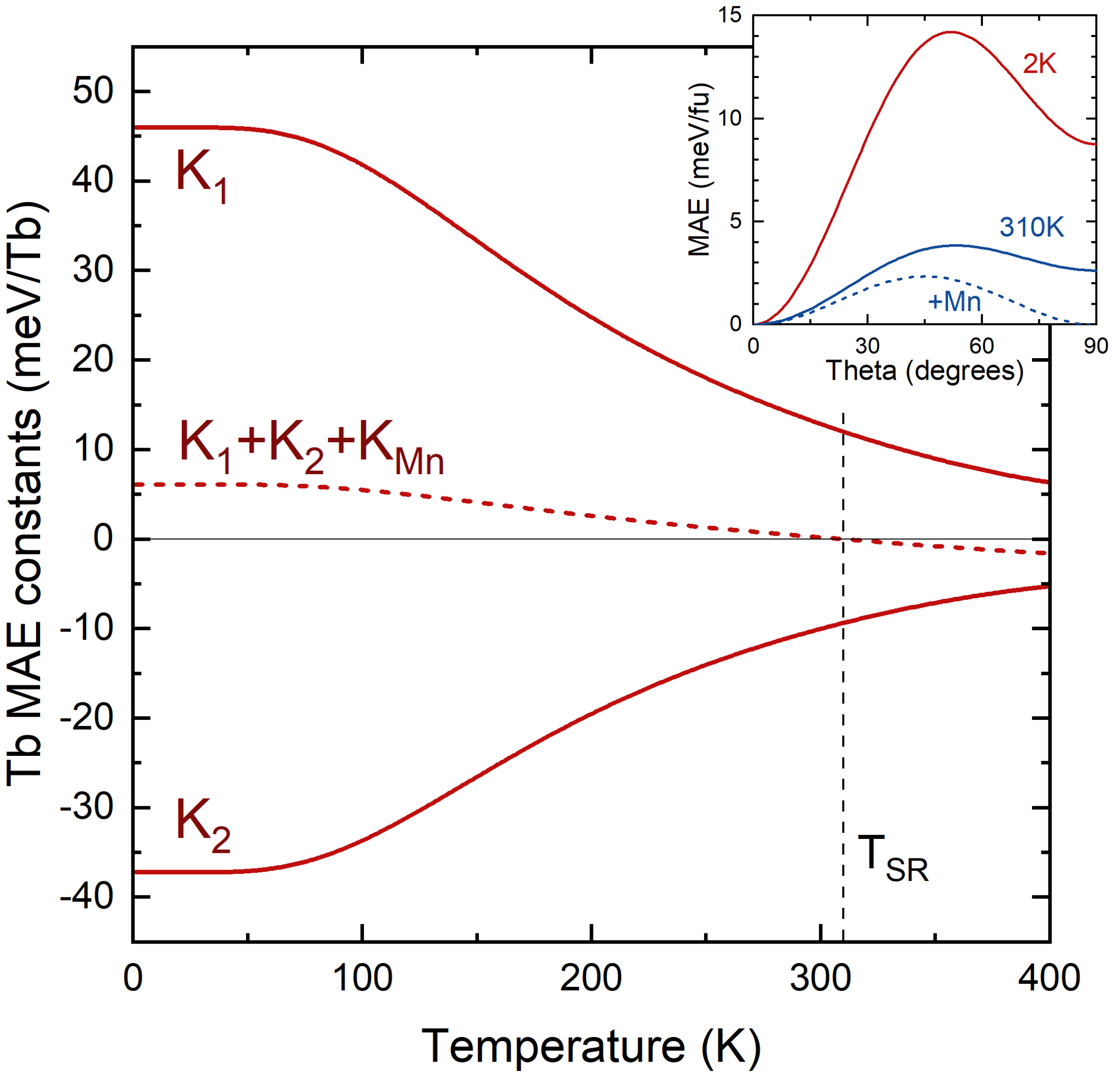

Supplementary Figure 4. **Calculated magnetic anisotropy constants of $TbMn_6Sn_6$.** The MAE constants for Tb as a function of temperature based on CEF parameters in Table 1 (solid lines). The dashed line shows the total MAE which crosses zero at T_{SR}. The inset shows the angular dependence of the MAE at 2 K (which is uniaxial in character) and at 310 K. Here, θ is the angle between the magnetic moment and the crystallographic *c*-axis. At 310 K, the inclusion of Mn easy-plane anisotropy (dashed line) results in two minima in the MAE for axial and easy-plane moment configurations.

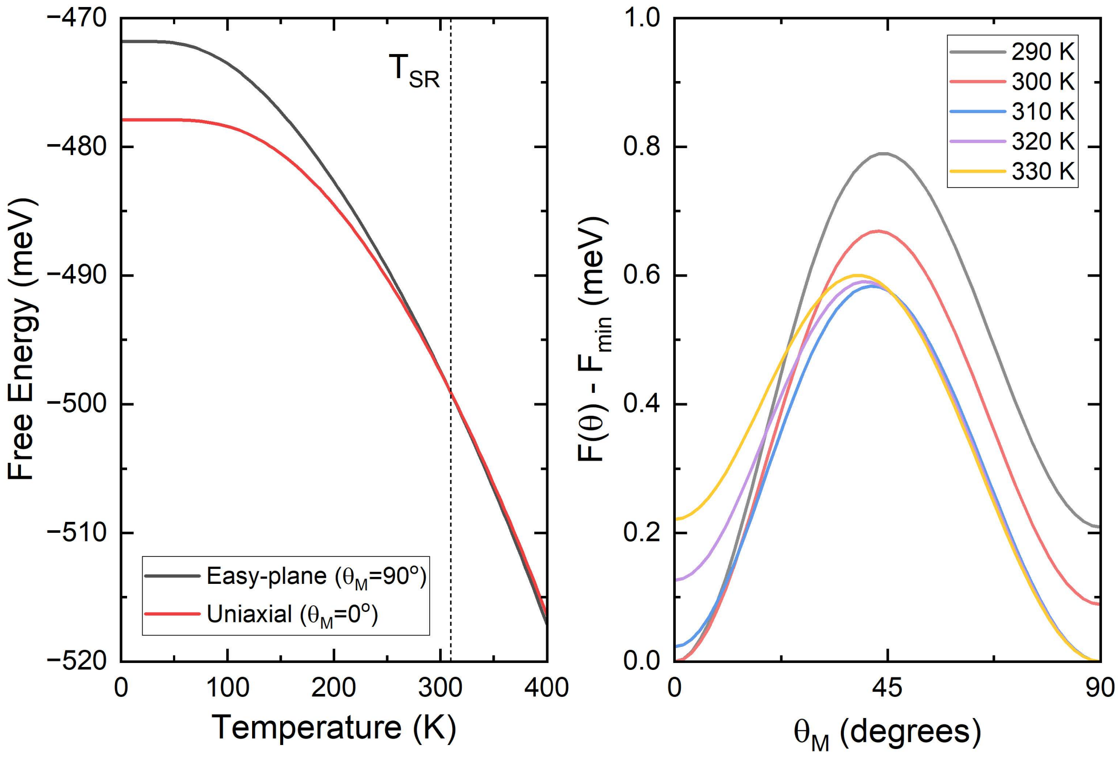

Supplementary Figure 5. **Free energy of $TbMn_6Sn_6$ in the mean-field approximation** (a) The free energy of the uniaxial and easy-plane states in Tb166 as a function of temperature. (b) The free energy minus F_{min} (the global minimum free energy) at various temperatures as a function of the angle of the Mn sublattice.
